# Surgical management of bilateral preaxial and postaxial polydactyly with syndactyly: A case report

**DOI:** 10.1016/j.ijscr.2024.110064

**Published:** 2024-07-20

**Authors:** Yoyos Dias Ismiarto, Mirna Phandu, Hans Kristian Handoko, Gregorius Thomas Prasetiyo, Fajri Rozi Kamaris, Tri Taufiqurachman Telaumbanua

**Affiliations:** a Division of Pediatrics Orthopaedic Surgery, Department of Orthopaedics and Traumatology, Faculty of Medicine Universitas Padjadjaran, Bandung, West Java, Indonesia; b Faculty of Medicine, Universitas Pelita Harapan, Tangerang, Indonesia; c Department of Orthopaedics and Traumatology, Faculty of Medicine Universitas Padjadjaran, Bandung, Indonesia

**Keywords:** Polydactyly, Syndactyly, Ablation, Z-plasty, Case report

## Abstract

**Introduction and importance:**

Polydactyly is the most common congenital malformation in the limbs. However, it is rare for a patient to exhibit concomitant preaxial and postaxial polydactyly alongside syndactyly in both limbs, and there are limited recommendations for such conditions. This report presents a case of bilateral preaxial and postaxial polydactyly with syndactyly of the feet.

**Presentation of case:**

A 2-year-old girl was presented with an excess number of toes on both feet and an abnormal connection between the second and third toes. After a physical examination and plain radiography, the patient was diagnosed with bilateral preaxial and postaxial polydactyly with syndactyly. We performed a one-stage surgical correction consisting of ablation at the extra digit of bilateral great and little toes, followed by syndactyly release using z-plasty. The surgery was uneventful, and the parents were satisfied with the result.

**Discussion:**

A plain radiograph is necessary for pre-operative planning. We discarded the excess digits for cosmetics and soft and hard tissue for optimal function. It is necessary to preserve the soft tissues, remove the auxiliary digit, realign the digit, and restore the ligaments to preserve digit stability.

**Conclusion:**

The complexity of this case required a meticulous surgical approach to address the structural abnormalities, restore functionality, and improve cosmetic appearance. In this case, the surgery can be performed in one procedure to minimize patient morbidity.

## Highlights

• It is rare for a patient to exhibit concomitant preaxial and postaxial polydactyly alongside syndactyly in both feet
• We performed a one-stage surgery involving the ablation of supernumerary digits, followed by syndactyly release using z-plasty
• We were not only discarding the excess finger for cosmetics but also discarding the soft tissue and hard tissue for optimal function of the feet

## 1 Introduction

Polydactyly and syndactyly are common congenital limb anomalies that can occur in isolation or concurrently. Polydactyly is the presence of extra digits on the hands or feet, while syndactyly is the fusion of two or more digits [1]. Polydactyly can be classified into postaxial, preaxial, and central ray duplication. Postaxial polydactyly is where the extra digit grows lateral to the fifth digit. It is the most prevalent polydactyly type, constituting around 80 % of reported instances. Preaxial polydactyly refers to the condition where the extra digit is positioned medially to the hallux. It is less prevalent, accounting for about 15 % of cases. Co-occurrent preaxial and postaxial polydactyly is rare and poses unique challenges for surgical intervention and functional rehabilitation. The incidence is rare and only reported in several case reports [2]. There are limited recommendations for such conditions. This report presents a case of bilateral preaxial and postaxial polydactyly with syndactyly of the feet in a tertiary hospital. The ethical commission of our university approved this case report. The patient's parents provided written informed consent. This case report was in line with SCARE 2023 criteria [3].

## 2 Case illustration

A 2-year-old Asian girl was presented to the pediatric orthopedic surgery polyclinic at a tertiary hospital with an excess number of toes on both feet and an abnormal connection between the second and third toes in both feet. The condition was present at birth. The patient was born with spontaneous vaginal delivery at normal gestational age. There was no history of trauma or family members having congenital disease. During pregnancy, there was no history of hypertension, premature membrane rupture, or bleeding. After birth, there were no vertebral defects, anal atresia, cardiac defects, tracheo-esophageal fistula, or renal anomalies (VACTERL). The patient had reached all developmental milestones. The nutritional status was normal according to z-score of BMI for age. The patient was right-handed.

**Fig. 1:**
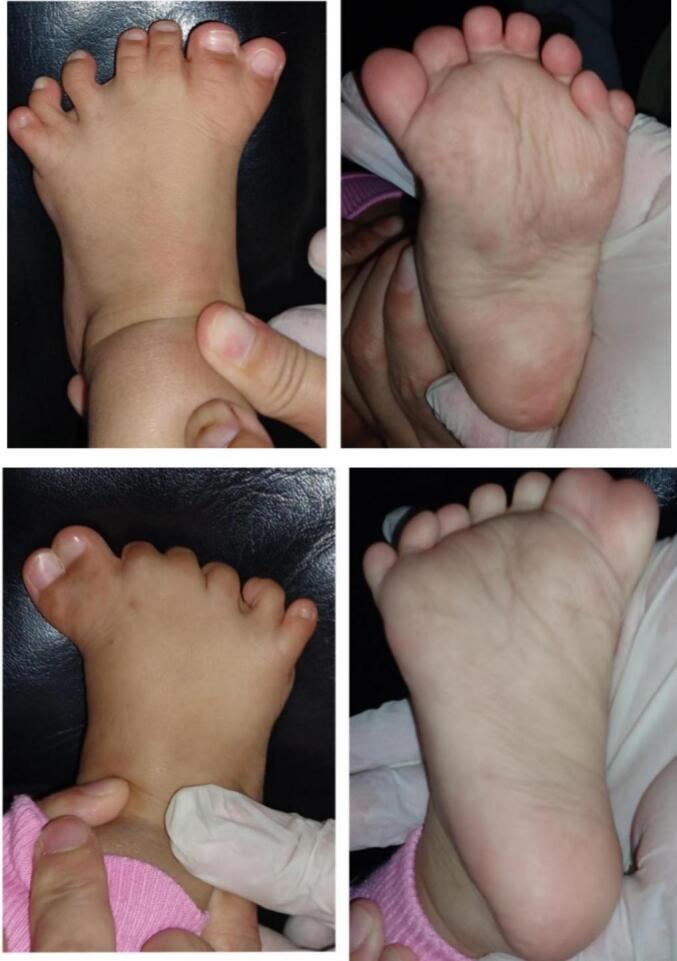
Preoperative photograph of a 2-year-old girl with bilateral preaxial and postaxial polydactyly with syndactyly of the feet.

**Fig. 2:**
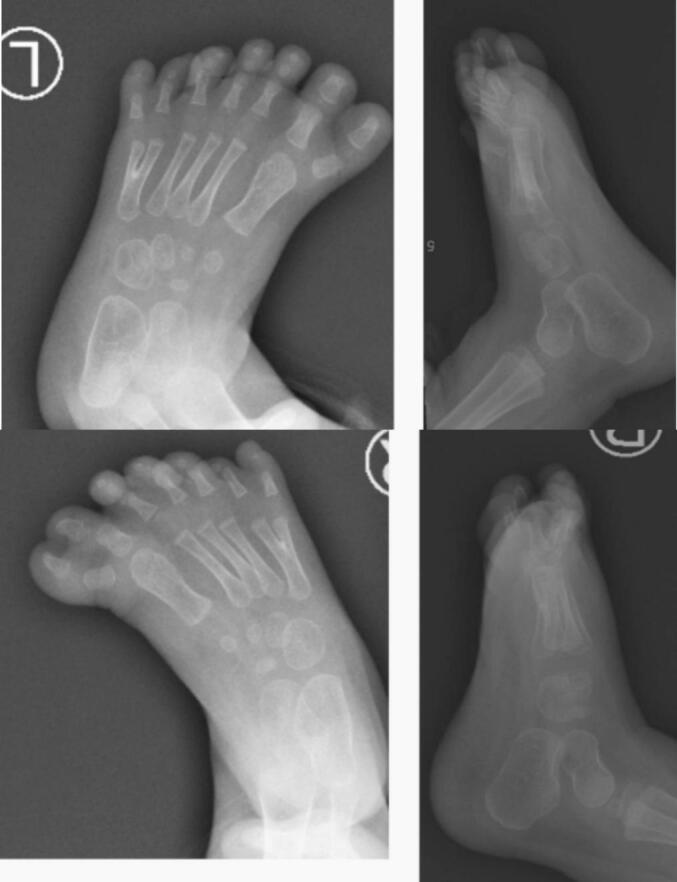
Anteroposterior and lateral radiographic examination of the left and right foot revealed a bifid fifth metatarsal.

Physical examination of the left and right feet revealed seven digits in each foot with constricted second and third digits. The range of motion was within normal limits (Fig. 1). Plain radiography revealed both feet's bifid fifth metatarsal bones (Fig. 2). The patient was diagnosed with bilateral preaxial and postaxial polydactyly with syndactyly. We then performed ablation of the extra digits at both great and little toes, followed by syndactyly release using z-plasty. The surgery was performed by an orthopedic surgeon and an anesthesiologist. We involved a pediatrician to evaluate the medical condition of the child and medical rehabilitation specialist for the rehabilitation.

## 3 Surgical technique

**Fig. 3:**
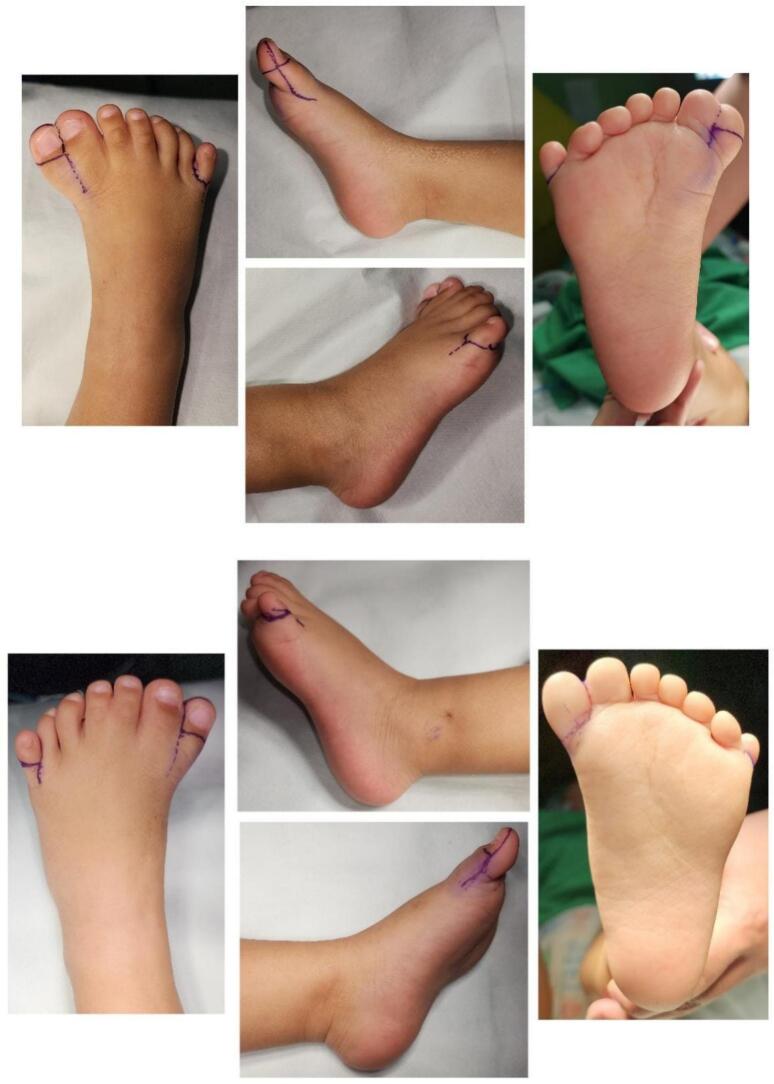
Surgical marking of bilateral preaxial and postaxial polydactyly with syndactyly.

**Fig. 4:**
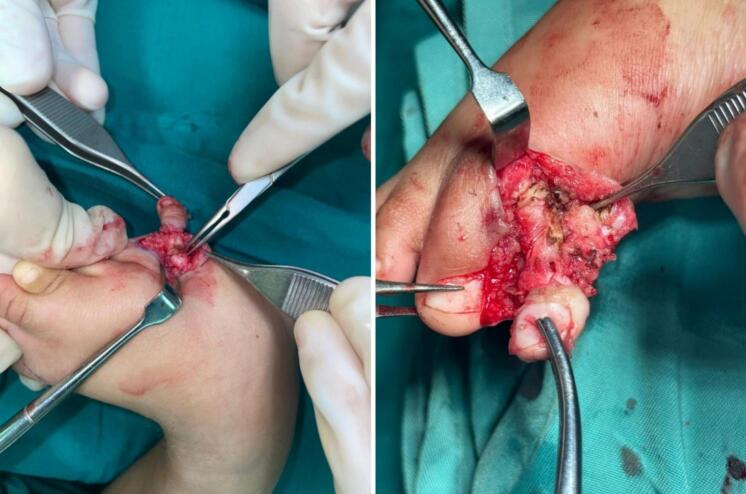
Intraoperative photograph of ablation at the extra digit of great and little toes.

**Fig. 5:**
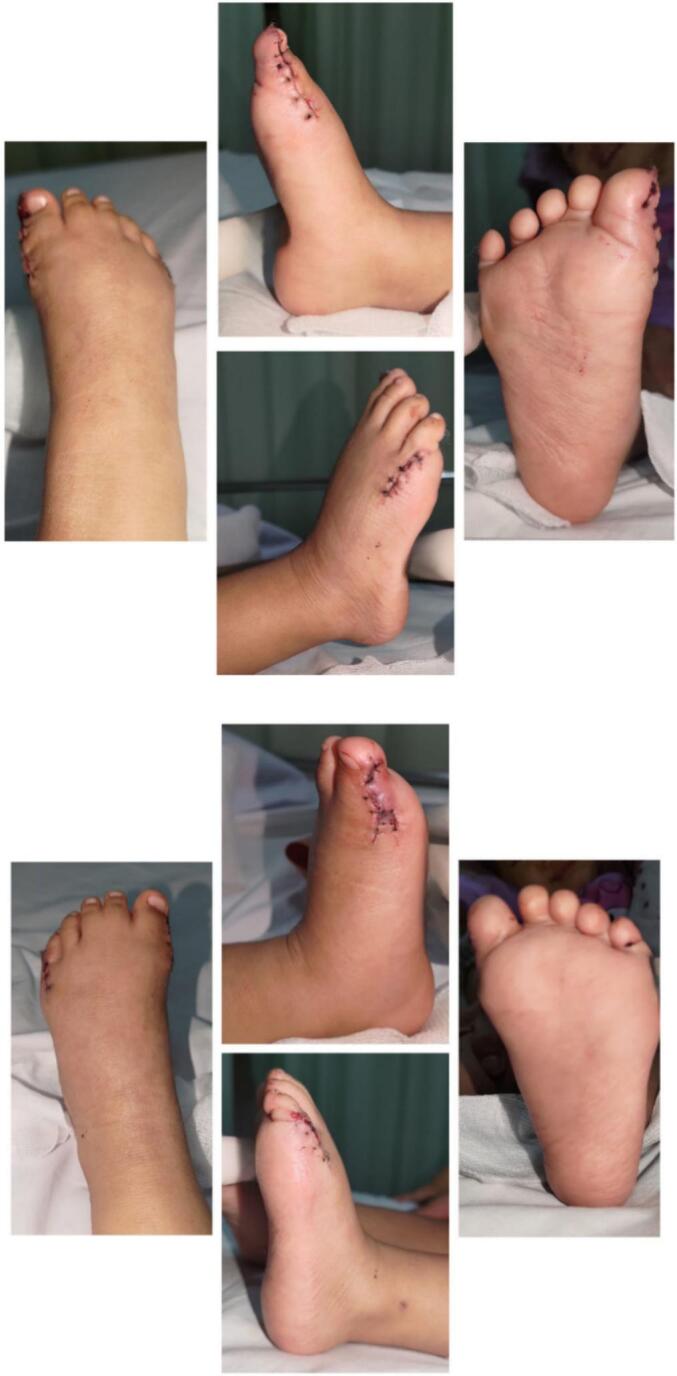
The photograph two-weeks after surgery.

The surgery was performed under general anesthesia, ensuring pain control and immobility of the patient. The procedure began with a precise incision following the preoperative markings (Fig. 3). The skin was carefully incised using a No. 15 blade scalpel, ensuring clean edges for optimal healing. Meticulous hemostasis was maintained throughout using bipolar electrocautery. Upon reaching the deeper layers, the subcutaneous tissue was carefully dissected using fine scissors and forceps to expose the deep fascia. A curved tenotomy scissor was then employed for blunt dissection of the deep fascia and tendons. This technique minimizes the risk of inadvertent damage to neurovascular structures. The extranumerary digits were carefully isolated, with particular attention paid to their neurovascular bundles. These structures were ligated using 4-0 silk sutures before the digits were resected to prevent excessive bleeding. The resection was performed at the appropriate level as determined in the preoperative planning (Fig. 4). Following digit removal, attention was turned to the bifid fifth metatarsal bone. Using fluoroscopic guidance to ensure precise placement, a small, sharp osteotome was utilized to perform an osteotomy. The osteotomy was angled to achieve proper alignment of the remaining metatarsal. Ligament and joint closure were meticulously performed using 5-0 Vicryl sutures in an interrupted fashion. This step is crucial for maintaining proper joint stability and alignment. Care was taken to achieve appropriate tension in the ligamentous structures. Finally, the skin was approximated and closed using simple interrupted sutures with 5-0 Prolene. This non-absorbable suture material was chosen for its strength and low tissue reactivity. The sutures were placed approximately 2–3 mm apart to ensure even distribution of tension along the wound edges (Fig. 5). Throughout the procedure, saline irrigation was used to keep the surgical site clean and maintain tissue hydration. A sterile dressing was applied post-closure. The physical therapy started one day after surgery, starting with gentle range of motion exercises to prevent stiffness. Weight-bearing is encouraged as tolerated, with close monitoring of the child's gait development.

The strength of this case report is presenting a rare and complex congenital hand anomaly. The article provides a thorough description of the patient's presentation, diagnostic workup, surgical approach, and outcomes. We also discuss the rationale behind performing a single-stage correction for both the polydactyly and syndactyly and the optimal timing for surgeries. However, The follow-up period described is relatively short (6 months), which limits the assessment of long-term outcomes and potential complications.

## 4 Outcome and follow-up

The surgery was uneventful, and the parents were satisfied with the result. The patient was discharged two days after the surgery. The suture was removed after 2 weeks (Fig. 5). Six months after surgery, the patient was pain-free. Clinical examination showed that the feet had a normal range of motion with no complications. The parents were confident about the patient's cosmetic appearance.

## 5 Discussion

Polydactyly is a congenital condition characterized by extra fingers or toes on the hands or feet. It is among the most common congenital limb anomalies and can range from a small, non-functional extra digit to a fully formed and functional digit. Polydactyly can occur as an isolated deformity or as part of a syndrome, and the severity and appearance vary widely among affected individuals. The incidence of polydactyly is 1–2 children out of 1000 live births [1]. Postaxial polydactyly is the most common type, while preaxial polydactyly only accounts for 15 % of cases [2]. Polydactyly is usually inherited in an autosomal dominant manner but can also manifest sporadically without family occurrences [[4], [5], [6]].

Syndactyly is a congenital disorder characterized by the fusion or webbing of two or more fingers or toes. During fetal development, the digits fail to separate properly, forming fused or webbed digits. This condition can vary in severity, with some cases involving only partial fusion while others involve complete fusion of the affected digits [7]. Syndactyly occurs in approximately 2–3 children per 10,000 live births [8].

Polydactyly and syndactyly are diagnosed by physical examination and plain radiography. Clinically, the patients may report discomfort when walking, trouble putting on shoes, psychological problems, and bad cosmetics [9]. The main principles in the surgical care of polydactyly include appropriate planning to obtain a straight, robust, functioning finger with minimal residual deformity. Available procedures include suture ligation, surgical ablation, advancement flap, z-plasty, and microsurgical flap [9]. Suture ligation is conducted when the extra digit doesn't contain bones [10]. Therefore, obtaining a plain radiograph is necessary in pre-operative planning. It is necessary to preserve the soft tissues, remove the auxiliary digit, realign the digit, and restore the ligaments to establish digit stability [4]. We discarded the excess toes for cosmetics and the soft and hard tissue for optimal function.

The timing for surgical correction of polydactyly and syndactyly varies across studies. Alzarmah et al. recommended that the surgery be conducted between 6 and 9 months to maximize the child's fine motor development [4]. Gonzalez et al. recommend surgical timing at 7–12 months, while Comer et al. recommend between 12 and 16 months [11,12]. Most experts agree that 12 to 18 months of age is optimal for surgery, considering the lower anesthetic risk. It is easier to reconstruct the larger digit at an age when it is early enough to catch fine motor milestones, and the patient is not yet aware of aesthetic appearance [13,14]. Performing surgery on a child that is too small may result in difficulty as they have small neurovascular anatomy, have a higher risk of vasospasm, and are less tolerant of anesthesia [2]. Surgery should also not be performed on children who are too old as it may disturb their motor development, and they have been aware of their aesthetic appearance, which may affect their psychology [15]. The surgery, in this case, was performed on a 2-year-old; the child has not realized their deformity and had normal developmental milestones.

The common complication after the correction of preaxial polydactyly is hallux varus [2]. A tendon transfer may be necessary to prevent this complication. Still, it was not conducted in this case as the tendon attachments on the remaining hallux were intact, with no clinically observed toe drift.

One-stage correction for syndactyly and polydactyly can be considered safe when performed under appropriate conditions and by experienced surgeons. This approach is often deemed safe because it reduces the overall number of surgical interventions and anesthetic exposures, which is particularly beneficial for young patients. Advanced surgical techniques and improved perioperative care have enhanced the safety profile of this complex procedure. The single-stage approach allows for a comprehensive correction of the hand's anatomy, potentially leading to better functional and aesthetic outcomes. It also minimizes the psychological impact on the child and family by avoiding multiple hospital stays and recovery periods. Additionally, performing both corrections simultaneously can lead to a more harmonious overall result, as the surgeon can address the interrelated aspects of syndactyly and polydactyly in a coordinated manner. However, it's crucial to note that the safety of this procedure heavily depends on careful patient selection, thorough preoperative planning, the surgeon's expertise, and the availability of appropriate postoperative care and rehabilitation services [16].Only limited studies report the *co*-occurrence of preaxial and postaxial polydactyly with syndactyly. The treatment was individualized depending on the patient anatomical condition. Kuah and Mollica performed preaxial polydactyly correction in a 10-month-old boy, postponing syndactyly release until the patient's skeletal maturity improved to reduce the risk of vasospasm [2]. In this case, the polydactyly and syndactyly surgeries were performed in one procedure to minimize patient morbidity, as the patient was already two years old. Alzarmah et al. reported using osteotome for bifid metatarsal bone and a small K-wire to realign the little finger in postaxial polydactyly in a nineteen-month-old girl [4]. A study in Nigeria performed preaxial polydactyly correction by extranumerary digit excision in a 2-month-old infant [17].

One-stage correction of bilateral preaxial and postaxial polydactyly with syndactyly is a complex procedure that requires careful planning and execution. To ensure success, surgeons should prioritize thorough preoperative assessment, including detailed imaging studies to understand the underlying skeletal and soft tissue anomalies. It's crucial to plan incisions meticulously to preserve blood supply and optimize aesthetic outcomes. During the procedure, careful dissection of neurovascular structures is important to maintain digit viability and sensibility. When addressing polydactyly, the surgeon must decide which digit to preserve based on functionality and appearance, often retaining the one with the best articular surface and tendon insertions. For syndactyly release, employing techniques like zig-zag incisions or V—Y advancements can help prevent scar contractures. Adequate soft tissue coverage is essential, and skin grafting may be necessary in some cases. Postoperatively, meticulous wound care and early limb rehabilitation are vital for optimal functional outcomes. Potential pitfalls include compromised blood supply leading to tissue necrosis, incomplete syndactyly release resulting in web creep, and suboptimal digit positioning affecting limb function. Surgeons should also be prepared for the increased operative time and the potential need for additional procedures as the child grows. Regular long-term follow-up is crucial to address any developing issues promptly [2,4,[7], [8], [9]].

## 6 Conclusion

Bilateral preaxial and postaxial polydactyly with syndactyly is a complex case that requires a meticulous surgical approach to address structural abnormalities, restore functionality, and improve cosmetic appearance. A plain radiograph is important to identify the osseous deformity. We performed one-stage correction by ablating the extra digit, followed by syndactyly release using z-plasty. In this case, the surgery can be performed in one procedure to minimize patient morbidity.

## Consent

The patient's parents/legal guardian received an explanation of the procedures and possible risks of the surgery and gave written informed consent. My manuscript does not contain any personal data. Written informed consent was obtained from the patient's parents/legal guardian for publication and any accompanying images. A copy of the written consent is available for review by the Editor-in-Chief of this journal on request.

## Ethical approval

This case report has been approved by the ethical commission of our university.

## Funding

This research did not receive any specific grant from funding agencies in the public, commercial, or not-for-profit sectors.

## Author contribution

Yoyos Dias Ismiarto: Surgeon, Conceptualization, Visualization, Methodology, Writing and Supervision.

Mirna Phandu: Writing and Supervision.

Hans Kristian Handoko: Surgeon and Writing.

Gregorius Thomas Prasetiyo: Surgeon and Writing.

Fajri Rozi Kamaris: Surgeon and Writing.

Tri Taufiqurachman Telaumbanua: Surgeon and Writing.

## Guarantor

The guarantor in this study is Yoyos Dias Ismiarto.

## Research registration number

This is not a First on Man study.

## Provenance and peer review

Not commissioned, externally peer-reviewed.

## Conflict of interest statement

The authors declare that there is no conflict of interest regarding the publication of this paper.
